# Decentering whiteness in libraries: a framework for inclusive collection management practices

**DOI:** 10.29173/jchla29815

**Published:** 2024-12-01

**Authors:** Caleb Nault

**Affiliations:** eCollections and Discovery Librarian University Health Network Toronto, ON, Canada

Jamison A. **Decentering whiteness in libraries: a framework for inclusive collection management practices**. Lanham, MD: Rowman & Littlefield; 2024. Hardcover: 143 p. ISBN: 978-1-5381-6290-3. Price: USD$98.00. Available from: https://rowman.com/ISBN/9781538162903/Decentering-Whiteness-in-Libraries-A-Framework-for-Inclusive-Collection-Management-Practices.

Amid book bans targeting material created by or featuring marginalized populations, drag queen story times interrupted by angry protests, and an ongoing overrepresentation of white individuals employed in libraries across Canada and the United States, social justice and equity remain crucial and challenging issues for the library profession. Efforts to address structural inequities in libraries are wide-ranging and take various forms, such as revising professional standards to incorporate diversity, equity, and inclusion (DEI) as principles, hiring with intentionality, and performing diversity audits on collections. In *Decentering whiteness in libraries: a framework for inclusive collection management practices*, Dr. Andrea Jamison makes a valuable contribution to the ongoing DEI movement, offering a practical guide to evaluating collection development policies for library professionals committed to fostering diversity, equity, and inclusion within their collections. Jamison, a scholar in school librarianship at Illinois State University, brings a wealth of experience and insight to this work. She has an extensive background in teaching and working with children and youth in school and library environments, and has been invited to speak internationally on topics including intellectual freedom, diversity and inclusion, and structural oppression in children’s books [1]. Jamison is also active in the American Library Association (ALA), where she chaired both the Ethnic & Multicultural Information Exchange Roundtable and the 2018 working group that revised ALA’s Library Bill of Rights for Diverse Library Collections [2]. *Decentering whiteness in libraries* is the culmination of her dissertation research assessing the extent to which diversity and inclusion were addressed in academic library collection development policies. In the preface, Jamison identifies the intended audience of the book as pre-service librarians who are interested in collection development (and more specifically, those planning on working with children and youth). The structure and design of the book reflects this intended audience, as it is written like a textbook with each chapter featuring clearly stated learning objectives, a summary of key words and main concepts, and discussion questions for further reflection and discussion. In a brief introduction, Jamison speaks to how her personal experiences of growing up in the American public education system as an economically disadvantaged and racialized child formed the impetus for writing the book.

The main content is organized into two parts. The first section contains three chapters, the first of which contextualizes why diversity and inclusion matter in libraries through an overview of the history of racism and ongoing landscape of inequality in American libraries. The second chapter provides a thorough background on the ALA's Library Bill of Rights and explains the significance of the document for helping libraries advocate for the rights of marginalized communities. In the third chapter, Jamison outlines collection management as an integral aspect of librarianship and articulates the benefits of having a written collection development policy.

The second part comprises five chapters that introduce practical tools, strategies, and recommended resources for library professionals to assess and improve their collection development policies. One of the standout features of the book is the introduction of the Jamison Measure of Diversity (J-MOD) assessment tool. Jamison designed this tool to help library workers evaluate their current collection development policies for the integration of diversity and inclusion language and subsequently identify areas that need improvement. The J-MOD is used to perform a content analysis of a policy, tracking the frequency of various words related to diversity, and providing a clear and quantifiable method of assessing the inclusivity of collection policies. Jamison provides a step-by-step guide for using the J-MOD and includes lists of synonyms for words relating to DEI, multiple case studies for understanding the application of the tool, and recommendations for how to write improved collection development policies after the evaluation is complete. The remaining chapters explore practical strategies and best practices for writing strong collection development policies that integrate diversity and inclusion, and include sample policies, tables, and lists of recommended resources which make *Decentering whiteness in libraries* a user-friendly guide for library professionals.

One of the book’s strengths is its comprehensive practical approach. Jamison both contextualizes why addressing social justice and diversity in libraries continues to be imperative for our profession and also offers actionable steps that library professionals can take to improve their collection development practices. Another strength is the structure and design of the text, which make for easy reference. The book is a quick read due to Jamison’s accessible and concise writing style along with the use of informative headings and bolded key terms, and the overall short length of 143 pages including recommended resources and an index. Library workers at any stage of their career are likely to find the book useful, particularly as it offers a stepped and measurable process for evaluating and writing library policy, which I am sure many can admit tends to fall by the wayside amid busy schedules.

The usual disclaimers to Canadian health and hospital library professionals are relevant here: the book is US-centric and refers to laws and histories that may need adjustment to be applicable in the Canadian context. Relatedly, Jamison writes for an audience of public, school, and academic library professionals; hospital library workers will need to adapt the J-MOD and other suggested tools to their environments. Further, readers who are looking for theory to support praxis may need to supplement this text with other publications. Despite its title, *Decentering whiteness in libraries* does not offer a definition of whiteness or explain what decentering whiteness in libraries means beyond evaluating policy for the inclusion of specific language. I found myself wishing for explicit links to theory and critical analyses of structural oppression as it relates to policy development in order to provide a fuller understanding of the significance of policy in social justice efforts in libraries.

Given the results of the 2024 CHLA/ABSC EDI Task Force membership survey, which found that only 13% of respondents “considered themselves as belonging to a visible minority group,” with 3% identifying as being of Indigenous ancestry, it is clear that DEI interventions are relevant in the Canadian health and hospital library landscape [3]. *Decentering whiteness in libraries* is a solid reference text for library professionals wanting to learn more about integrating DEI practices into collection management overall, and especially for those who desire an easy, actionable guide to assessing and reworking existing policies or creating new ones.
